# Eye-tracking analysis of attentional disengagement in phobic and non-phobic individuals

**DOI:** 10.3758/s13414-024-02968-6

**Published:** 2024-10-10

**Authors:** Christina Saalwirth, Maximilian Stefani, Marian Sauter, Wolfgang Mack

**Affiliations:** 1 Department of Human Sciences, Institute of Psychology, General Psychology, University of the Bundeswehr Munich, Werner-Heisenberg-Weg 39, 85577 Neubiberg, Germany; 2 Institute of Psychology, General Psychology, Ulm University, Ulm, Germany

**Keywords:** Attentional disengagement, Visual search, Behavioral freezing, Fear, Phobia

## Abstract

This study investigated threat-related attention biases using a new visual search paradigm with eye tracking, which allows for measuring attentional disengagement in isolation. This is crucial as previous studies have been unable to distinguish between engagement, disengagement, and behavioral freezing. Thirty-three participants (*M*_age_ = 28.75 years, *SD* = 8.98; 21 women) with self-reported specific phobia (spiders, snakes, and pointed objects) and their matched controls (*M*_age_ = 28.38 years, *SD* = 8.66; 21 women) took part in the experiment. The participants were instructed to initially focus on a picture in the center of the screen, then search for a target picture in an outer circle consisting of six images, and respond via a button press whether the object in the target picture was oriented to the left or right. We found that phobic individuals show delayed disengagement and slower decision times compared with non-phobic individuals, regardless of whether the stimulus was threat-related or neutral. These results indicate that phobic individuals tend to exhibit poorer attentional control mechanisms and problems inhibiting irrelevant information. We also confirmed a threat-unrelated *shared feature effect* with complex stimuli (delayed disengagement when an attended stimulus and an unattended target share common stimulus features). This process might play a role in various experimental setups investigating attentional disengagement that has not yet been considered. These findings are important, as good attentional control may serve as a protective mechanism against anxiety disorders.

## Introduction

The dominant emotions associated with specific phobias are fear and anxiety, which serve as a critical mechanism, facilitating the identification of potential threats and thereby playing a pivotal role in survival (LeDoux, 1996). Nonetheless, aberrations in responses to such threatening stimuli could be essential in the pathogenesis and persistence of anxiety-related disorders as maladaptive manifestations of anxiety, such as heightened attention to non-threatening stimuli, may result in anxiety disorders such as specific phobias (Eysenck, 1997; Eysenck et al., 2007).

Over the past four decades, numerous studies have investigated threat-related attentional bias in anxious and non-anxious individuals (exhibiting social phobia, obsessive-compulsive disorder, generalized anxiety disorder, or specific phobias). These studies have posited that altered spatial attentional processes occur in the presence of threat-related stimuli (see meta-analysis by Bar-Haim et al., 2007; Clauss et al., 2022). However, one has to differentiate between different components of spatial attention. According to Posner (1980), spatial attention can be decomposed into engagement, disengagement, and shifting. Engagement is defined as initiating attentional processing, encompassing saccade planning, wherein attention is directed towards a stimulus. Disengagement signifies the cessation of processing, involving the withdrawal of attention from a stimulus, which will be the primary focus of this investigation. Shifting, the final component, pertains to the transition of attention toward a novel stimulus.

Differentiating which of these specific processes are altered in threat-related attentional bias is important to get a better understanding of anxiety disorders and to be able to improve treatment methods. Existing research has identified biases in attentional engagement (faster engagement) and disengagement (delayed disengagement; Bar-Haim et al., 2007). However, the efficacy of current experimental paradigms in differentiating these processes remains a subject of ongoing debate (see, e.g., Armstrong & Olatunji, 2012; Clarke et al., 2013). In this regard, this study aims to introduce a new way of investigating attention bias with a novel experimental design that is able to examine attentional disengagement processes in isolation.

### Measuring threat-related attentional bias

Two primary methodologies, *covert* and *overt attention*, have been employed in the past to investigate attentional processes, each with unique advantages and limitations. When inferences are derived through the comparative analysis of different experimental conditions, it is referred to as *covert attention*, as the attentional allocation remains unobservable. Experimental paradigms such as the dot-probe task (MacLeod et al., 1986) or the spatial cueing task (Fox et al., 2001; Koster et al., 2006; Posner, 1980) have been extensively employed to explore threat-related attentional bias (Bar-Haim et al., 2007; Clauss et al., 2022).

The dot-probe task (e.g., Grafton & MacLeod, 2014; MacLeod et al., 1986) measures selective attention and attentional biases, particularly the speed of response to threatening versus neutral stimuli. The spatial cueing task (e.g., Fox et al., 2001; Mogg et al., 2008) is a paradigm for studying visual attention where attention is drawn to a specific location due to the appearance of a stimulus. It demonstrates that we are quicker to detect objects in places that have been cued before. In these tasks, attentional engagement is usually measured as the latency of the response to a stimulus when a threatening cue is presented in the attended location. In contrast, attentional disengagement is measured as the latency of the response to a threatening stimulus when a threatening cue is presented in an unattended location. Fox et al. (2001) utilized a modified version of this spatial-cueing paradigm to include affectively valenced cues to assess different patterns of disengagement in high- and low-anxious individuals. The key finding was that high-anxious individuals took longer to disengage their attention from threat-related cues than low-anxious individuals, indicating difficulty in shifting attention away from threats. However, it has been demonstrated that both tasks have low reliability due to their methods of measuring attention processes (Chapman et al., 2019; McNally, 2019) and only capture attention within a small time window, likely after several shifts in attention have already occurred (Mogg & Bradley, 1998).

Furthermore, Mogg et al. (2008) challenged the interpretations of the attentional processes within the spatial cueing task, arguing that the observed delayed disengagement might be influenced by factors such as a general slowing of responses or behavioral freezing because participants in their experiment showed slower responses to threat-related stimuli when no deployment of attention was necessary. Behavioral freezing can occur in the presence of a threat and is characterized by a sudden and temporary cessation of movement and a heightened state of alertness while the individual assesses the situation and decides on the appropriate response (Hagenaars et al., 2014). Behavioral freezing occurs only when an individual is uncertain about their future actions, specifically whether to flee or fight (orientation reaction). While the dot-probe task was employed to mitigate the influence of behavioral freezing by simultaneously presenting a neutral and a threatening stimulus (MacLeod et al., 1986), participants in this paradigm do not need to attend to the threatening stimuli, which appear at a distance to the point of fixation. This means it remains unclear whether the threatening stimulus was attended to or not. Therefore, the question remains about which role behavioral freezing plays in attentional bias to threat.

In contrast, complementary to covert attention measures, a second approach measures *overt attention* via eye tracking (e.g., Armstrong & Olatunji, 2012; Clauss et al., 2022; Sanchez et al., 2013). Eye tracking can record eye movements, such as fixations or saccades, allowing conclusions about different attentional processes. Here, (oculomotor) engagement refers to the latency of the first shift in gaze from a neutral to a threatening stimulus, and (oculomotor) disengagement refers to the latency of the first shift in gaze away from a threatening stimulus to a neutral stimulus. An advantage of eye tracking, as Armstrong and Olatunji (2012) argue, is that eye movements are less susceptible to confounding information processes and do not appear to be affected by behavioral freezing (McNaughton & Corr, 2004; Nummenmaa et al., 2006). However, attention resources could possibly be shifted without any saccadic eye movements (Armstrong & Olatunji, 2012), and as Sagliano et al. (2016) pointed out, how precisely *overt* and *covert attention* relate to attentional engagement and disengagement processes is not fully understood yet. Therefore, a joint measuring of *overt* and *covert attention* seems to be the best option to investigate anxiety-related attentional bias (see also the meta-analysis by Clauss et al., 2022, about measuring threat-related attentional bias with covert and overt attention). Thus, with our proposed experimental design using eye tracking, we included both overt and covert measures of attention (for more information, see the section titled “The Experimental Design”).

So far, for *covert* and *overt attention*, two general types of tasks have been used to investigate threat-related attentional bias—free viewing and visual search tasks. These tasks revealed different altered attentional processes when investigated with eye tracking. Armstrong and Olatunji (2012) conducted a comprehensive review of studies employing eye-tracking methodologies to investigate attentional biases in anxiety (see also Clauss et al., 2022). Their analysis revealed that anxious individuals tend to exhibit facilitated attentional engagement with threat-related stimuli in free-viewing tasks. This means that when participants freely view a scene, they are quicker to fixate on threatening stimuli than neutral stimuli, indicating a heightened vigilance towards potential threats. In contrast, visual search tasks, which require participants to locate a target stimulus among distractors, revealed a different pattern. Specifically, these studies found that anxious individuals experience delayed disengagement from threat-related stimuli. This means that once a threatening stimulus captures their attention, they have difficulty shifting their gaze away to find the target stimulus. Interestingly, these tasks did not show significant differences in the initial engagement with threat-related stimuli, suggesting that the primary attentional bias in visual search tasks is related to disengagement rather than engagement. Therefore, detecting threat-related attention bias seems dependent on the experimental paradigm, and delayed disengagement effects are best investigated with visual search tasks, yet most of the studies using eye tracking to investigate attentional bias for threat-related stimuli have been implementing free-viewing tasks (see, e.g., Nelson et al., 2015; Quigley et al., 2012).

### Underlying mechanisms

**Fig. 1:**
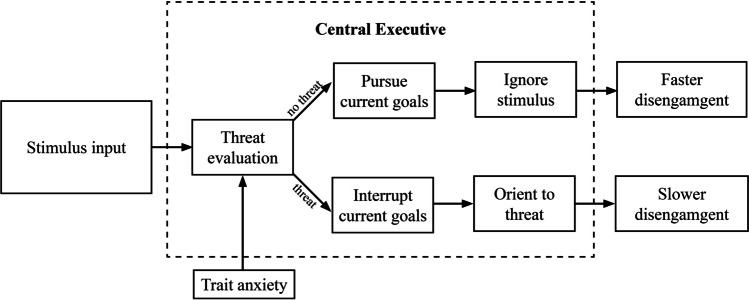
Threat-related stimuli influence processes in the central executive (inhibition, shifting, and updating). Specifically, the inhibition of threat-related stimuli in participants with high trait anxiety is impaired. Adapted from Chow and Mercado (2020)

Although numerous research studies have documented a threat-related attention bias for engagement and disengagement processes, the underlying mechanisms are not fully understood. If we want to explain these altered processes, specifically delayed disengagement, in the presence of threat-related stimuli, the Attentional Control Theory (ACT; Eysenck et al., 2007) can significantly contribute. The ACT posits three central executive functions in controlling attention: inhibition, shifting, and updating. In particular, the role of inhibition appears to be important, as individuals with high levels of anxiety exhibit difficulty in ignoring irrelevant information, highlighting the involvement of the central executive (see Fig. 1).

The central executive involves both bottom-up and top-down processes. While the top-down system focuses on goal-oriented and context processing and is closely related to activity in the prefrontal brain regions, the bottom-up system focuses on stimulus-driven information processing (physical characteristics and appraisal of emotions) and is associated with amygdala-based mechanisms (Corbetta & Shulman, 2002; Sussman et al., 2016). According to the ACT, anxiety has a dual effect on attentional processes in anxious individuals (see Shi et al., 2019). Firstly, it enhances the stimulus-driven bottom-up attention system, which leads to increased sensitivity and faster detection of threatening stimuli in the environment. Secondly, anxiety impairs the top-down mechanisms of goal-directed attentional control, as hypervigilance, due to the anticipation of adverse future events, may lead to deficiencies in working memory capacities (Sussman et al., 2016). This dual effect leads to increased allocation of attention to potentially threatening stimuli while at the same time reducing the efficiency of voluntary attentional control processes (Eysenck et al., 2007). Consequently, non-threatening stimuli are also processed more inefficiently, which could be observed in accuracy scores (Calvo et al., 1994; Ikeda et al., 1996) and reaction times (Bishop et al., 2004; Compton et al., 2003). However, it is difficult to adequately differentiate between the influence of top-down or bottom-up processes, as the two effects constantly interact (Sussman et al., 2016).

### Isolating attentional processes

**Fig. 2:**
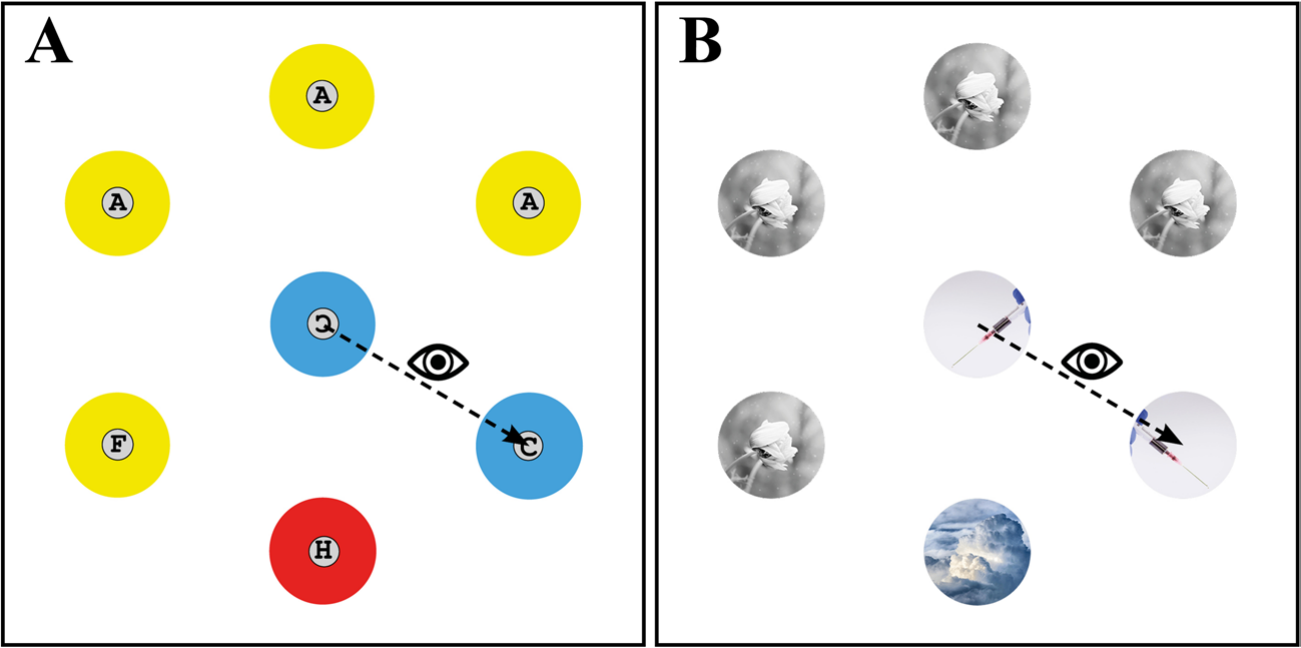
Search display **A** shows the visual search task in a non-threat-related context. The participant must start from the center to find the blue target in the peripheral circle and decide in which direction “C” is oriented. Search display **B** shows the visual search task in a threat-related context (phobia towards pointed objects). The task for the participant is the same as in search **A**, except that now a specific picture must always be searched for. (Color figure online)

In studies on attention bias without context anxiety, it has been possible to isolate individual processes (engagement, disengagement, and shifting) and to examine the effects of top-down and bottom-up on dwell times on certain stimuli with eye tracking. For example, with the visual search paradigm from Stefani et al. (2020), isolating disengagement processes while keeping the engagement and shifting process constant across conditions is possible. From contingent capture studies, we know that unexpectedly appearing irrelevant color singletons (distractors) attract attention. They not only capture attention but also delay attentional disengagement. However, it has been shown that bottom-up salience alone is insufficient and that the delay in attention is strongly influenced by top-down mechanisms (Born et al., 2011). Boot and Brockmole (2010) and later Stefani et al. (2020) were able to show that when participants were instructed to shift their gaze (saccade) from a central and irrelevant object to a specific-colored target among several objects arranged in a circle, disengagement was constantly delayed if the central fixation shared features with the search target (e.g., the color). We will refer to this effect as the *shared-feature effect* (see Fig. 2A). Even when the irrelevant center object did not exactly match the search target’s features, a constant delay in attention could be observed. This must be related to top-down mechanisms because the task goal leads to a deeper processing of irrelevant stimuli (Blakely et al., 2012; Stefani et al., 2020; Wright, Boot, & Brockmole, 2015a, Wright, Boot, & Jones, 2015b).

When considering the effects of threat-related stimuli on anxious individuals, the shared-feature effect is likely to be significantly amplified due to impairments in top-down processing. This means that not only do irrelevant stimuli capture their attention but they also struggle to suppress the (irrelevant) threat-related stimuli. This amplification manifests in both the engagement and disengagement of attention (see also Koster et al., 2005), resulting in slower disengagement from threat-related stimuli in visual search paradigms (see Fig. 2B for a sample search with threat-related stimuli—here, pointed objects). Notably, Bishop (2009) found that even in the absence of threat-related stimuli, high-trait-anxiety individuals exhibit diminished attentional control in the prefrontal cortex, suggesting a generally reduced ability to inhibit task-irrelevant distractors. Despite prolonged disengagement from threatening stimuli, Derakshan and Koster (2010) observed similar error rates between anxious and non-anxious individuals in antisaccade tasks. This finding aligns with Armstrong and Olatunji’s (2012) assertion that anxious individuals may be equally effective but less efficient when disengaging from threats. These results further support the ACT, highlighting that while anxious individuals struggle to inhibit initial threat processing, they may employ compensatory strategies to maintain performance effectiveness. This pattern underscores the complex interplay between bottom-up, stimulus-driven attentional processes and top-down control mechanisms in anxiety, where heightened sensitivity to threats coexists with impaired voluntary attentional control. It is, therefore, necessary to keep as many factors as possible constant in experimental designs.

### Disgust in specific phobia

When investigating attentional biases in specific phobias, one also has to account for the role of disgust. Besides the primary emotions of fear and anxiety, disgust is consistently associated with certain types of specific phobias, such as spider, snake, or blood-injection-injury phobia, while the specific mechanisms still remain unclear (Knowles et al., 2019; Tolin et al., 1997). Disgust, like fear and anxiety, is a negatively valenced emotion that may motivate people to avoid disease and contamination from encounters with spiders, snakes, or blood (Matchett & Davey, 1991). However, since disgust is also associated with psychological disorders that do not involve disease avoidance, this explanation seems, at least in part, insufficient (Knowles et al., 2019). Nevertheless, given our primary focus on threat-related attentional biases arising from the primary emotional reactions in specific phobias—namely, fear and anxiety—it is crucial to incorporate disgust as a control variable. By doing so, we can better isolate the effects of fear and anxiety and gain a more comprehensive understanding of the underlying mechanisms.

### The experimental design

The present study aims to address the limitations of previous research in distinguishing between attentional processes, such as disengagement or engagement, and other behavioral processes. Prior studies that measured mostly covert attention could not accurately differentiate these processes as their conclusions were based solely on mean differences calculated between various conditions. Similarly, studies that measured overt attention failed to isolate specific attentional processes from one another, leading to the same issue of indistinguishable attentional processes. In addition, most studies failed to control for potential behavioral freezing (Clarke et al., 2013).

We can overcome these limitations with an adapted version of the visual search paradigm from Stefani et al. (2020) by paying attention to the following three aspects: (1) maintaining a fixed starting position for the eye (always in the center of the search display), which ensures a consistent disengagement in a neutral search; (2) keeping the engagement constant across all trials by providing a constant instruction to search for a specific target (search for a predefined picture in an experimental block; this eliminates any uncertainty about whether to shift attention or not; and (3) keeping a constant distance between the search targets and the starting position, which ensures that the shifting process remains uniform (cf. Clarke et al., 2013; Mogg et al., 2008; see section 2.3.1 for more details).

Furthermore, our study combines covert and overt attention measures to provide further insight into anxiety-related attentional bias while controlling for behavioral freezing during the disengagement process. The participants were instructed to ignore the start stimulus and to move their attention directly to the target. Thus, there should be no uncertainty about the appropriate action. We included participants with and without an object-related self-reported specific phobia (towards mice, dogs, snakes, spiders, pointed objects, and dentists), as this anxiety disorder allows for an easy visual representation of the threat-inducing stimuli. It is also characterized by persistent and excessive anxiety about a certain object or situation where individuals disproportionately focus on potentially threatening information regarding this object or situation (Bar-Haim et al., 2007). Although attentional biases occur in all anxiety disorders, specific phobias offer a clear and straightforward way to study these biases due to the well-defined nature of the threatening triggers (Bar-Haim et al., 2007).

### Aims of the study

**Fig. 3:**
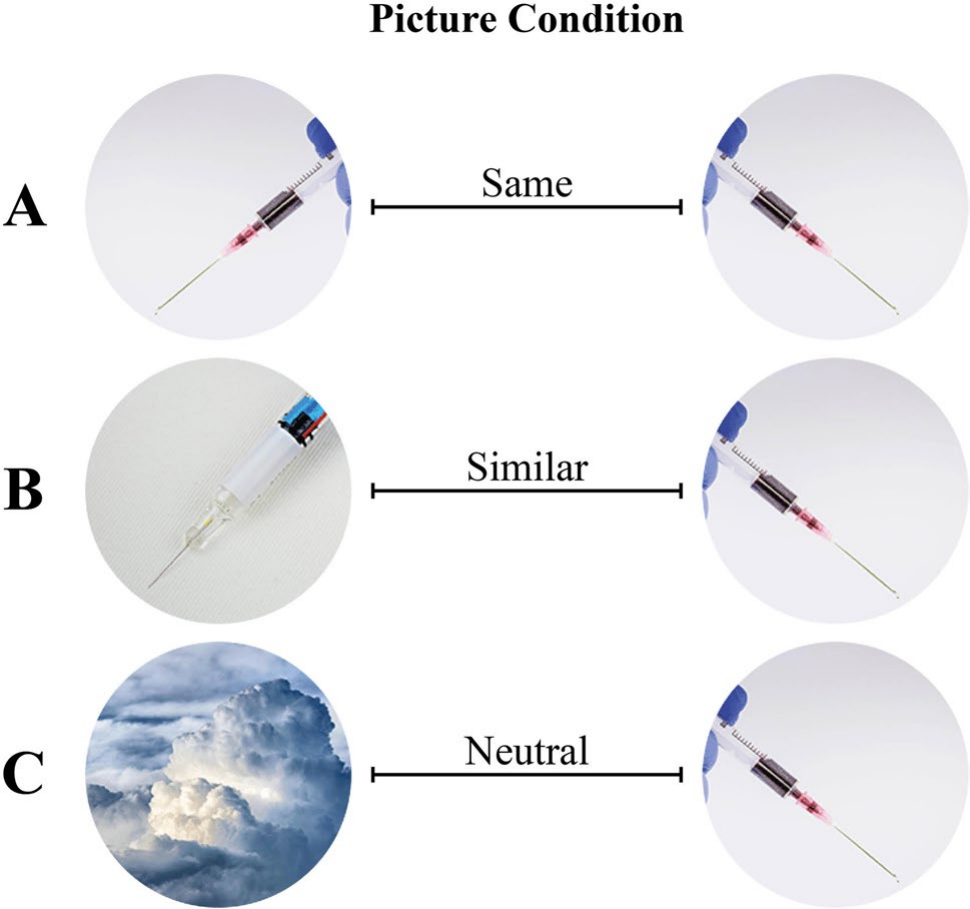
Examples of the same (A), similar (B), and neutral (C) picture conditions for pointed objects

With this study, we aim to introduce a newly adopted experimental approach to investigate attention bias with eye tracking. Using this adopted experimental design, we first want to replicate the so-called shared-feature effect found in the original experimental paradigm for complex visual stimuli (see H1 and H1a). The shared-feature effect, which is independent of threat or specific phobia, occurs when a start picture shares features with a target picture, resulting in delayed disengagement (Stefani et al., 2020). We hypothesize that the “same” picture condition (start and target picture are the same; see Fig. 3A) will reveal slower saccadic latencies than the “similar” (start and target picture are similar; see Fig. 3B) and “neutral” picture conditions (start and target picture are unrelated; see Fig. 3C) because more features are shared in the same compared with the similar and neutral picture conditions, respectively.

Secondly, we further aim to test for a threat-related delayed disengagement of attention in individuals, with and without self-reported specific phobia, towards mice, dogs, snakes, spiders, pointed objects, and dentists representing the most common object-related types of specific phobia. We expect slower saccadic latencies for participants with self-reported phobia (phobic group) compared with participants without self-reported phobia (non-phobic group), independent of the picture condition (see H2). We expect this difference to be driven by slower saccadic latencies (delayed disengagement) when phobic individuals, compared with non-phobic individuals, see a threat-related picture (same and similar picture conditions) in their initial locus of attention (see H3, H3b, and H3c). No differences between phobic and non-phobic individuals for neutral pictures should occur (see H3a). Lastly, we expect slower decision times for the phobic group regarding the target picture orientation (left or right) due to poorer inhibition of the phobic compared with the non-phobic group (see H4). These research aims led to the following hypotheses:

- H1: There is a shared feature effect (slower saccadic latencies) in the same and similar picture conditions compared with the neutral picture condition, independent of self-reported phobia.
- H1a: The shared feature effect (slower saccadic latencies) in the same picture condition is stronger than in the similar picture condition.
- H2: Saccadic latencies for participants in the phobic group are slower compared with participants in the non-phobic group.
- H3: There is an interaction effect between group (phobic vs. non-phobic) and picture condition (same, similar, neutral).
- H3a: In the neutral picture condition, the phobic and non-phobic groups’ saccadic latencies (delayed disengagement) do not differ.
- H3b: In the same picture condition, the phobic group’s saccadic latencies (delayed disengagement) are slower than in the non-phobic group.
- H3c: In the similar picture condition, the phobic group’s saccadic latencies (delayed disengagement) are slower than in the non-phobic group.
- H4: The phobic group’s decision time is slower compared with the non-phobic group.

In conclusion, this study aims to provide a more nuanced understanding of attentional processes in the context of threat-related attentional bias. By adopting a novel experimental approach, we hope to effectively control for potential confounding factors, such as behavioral freezing, and provide a more accurate differentiation between engagement and disengagement processes.

## Methods

### Participants

We distributed an online questionnaire via email at the University of the Bundeswehr Munich to find participants who met our experiment’s inclusion criteria. At the beginning of the questionnaire, participants were automatically excluded if they were currently, in the past, or planned to be under professional treatment for anxiety disorders and were under 18 years of age. A total of 314 participants completed the questionnaire.

Based on the answers in the questionnaire, participants scoring below 3.0 or above 5.0 on the adapted Fear of Spiders Screening scales (see the section titled “Online Questionnaire”) were allocated to the non-phobic and phobic groups, respectively. Those falling within the range of 3.0 to 5.0 were classified into the medium-phobic group and were excluded. The phobic group was further divided into six subgroups according to the reported type of specific phobia (fear of mice, dogs, snakes, spiders, pointed objects, and dentists). Participants who reported multiple types of specific phobias were randomly assigned to a subgroup. Because we defined the minimum number of participants per subgroup to ten, the specific phobia conditions of dogs, mice, and dentists were excluded. Each participant in the phobic group was matched with a participant in the non-phobic group regarding age (±2 years), gender (man or woman), and type of phobia. The same objects were presented to the participant in the phobic group and its counterpart in the non-phobic group. Age and gender were chosen as control variables because reaction times significantly increase with age (Deary & Der, 2005), and women show a higher prevalence of anxiety disorders in general (McLean et al., 2011).

Participants of the phobic group (*N* = 55) who had a potential match in the non-phobic group were invited to participate in the experiment. After the participants of the phobic group completed the experiment, their matched controls were also invited to participate. Two participants of the phobic group were excluded (and therefore also their counterparts in the non-phobic group) because they were defined as outliers since they showed reaction times that were three standard deviations above the mean in their age group (Tabachnick et al., 2013).

The final study sample consisted of 33 participants in the phobic group (*N* = 21 women, *M*_age_ = 28.75 years, *SD* = 8.98) and 33 participants in the non-phobic group (*N* = 21 women, *M*_age_ = 28.38 years, *SD* = 8.66). All participants that underwent the experiment had a normal or corrected-to-normal vision and were compensated for their participation with 20 euros and course credit if applicable. All participants gave informed consent, and the study was approved by the Ethics Committee of the University of the Bundeswehr Munich.

To measure disengagement using saccadic latencies, a power analysis was conducted a priori using the software program G*Power for repeated-measures analyses of variance (ANOVAs). The analysis included a between-subject factor for the group (phobic and non-phobic) and a within-subject factor for picture condition (similar, same, neutral). Based on the results of Stefani et al. (2020), yet more restrictive, we assumed a medium effect size *f* of .25. We aimed for a significance level (α) of .05 and a statistical power (1 − β) exceeding 0.95 (Faul et al., 2009). The power analysis suggested a total sample size of *N* = 44.

### Online questionnaire

The online questionnaire consisted of questions about the participants’ social demographics (age and gender); psychotherapeutic treatment of anxiety disorders (yes or no); existing phobia towards mice, dogs, snakes, spiders, pointed objects, or dentist (yes or no); and the intensity of the corresponding phobia which was measured with an adaption of the Fear of Spiders Screening by Rinck et al. (2002). The Fear of Spiders Screening was specifically developed to identify people with spider phobia in large populations in a parsimonious way and offers good reliability and validity (Rinck et al., 2002). Further, the four items of the Fear of Spiders Screening correspond to the four relevant criteria of the *Diagnostic and Statistical Manual of Mental Disorders* (DSM-IV; American Psychiatric Association, 1997) diagnosis of “Specific Phobia,” thus offering a high level of content congruency. Because we did not find a questionnaire assessing all these types of specific phobias in a parsimonious way, we replaced the word *spider* with the other objects of specific phobias we wanted to assess. The four items for each type of specific phobia (7-point Likert scale) were aggregated into a mean score, with higher scores indicating stronger phobia. Reliability was good for the three types of specific phobias (snakes, spiders, pointed objects) that were included in the study (Cronbach’s α = .94–.98), and the manipulation check conducted in this study indicated good validity (see the section titled “Manipulation Check”).

### Experimental procedure

In the laboratory, the participants were first informed about the upcoming experiment’s procedure. Next, the pictures presented in the experiment, depending on the phobia subgroup, were presented. If the participants felt able to perform the experiment, they then rated the pictures regarding their intensity levels of fear and disgust with one item each on a 7-point Likert scale (1 = *no fear/disgust at all* to 7 = *very strong fear/disgust*), with higher scores indicating stronger fear/disgust. We measured disgust as a control variable since some types of phobia, such as phobia towards snakes and spiders, can also trigger a feeling of disgust in addition to fear. This is relevant as disgust is associated with attentional biases on its own (Charash & McKay, 2002; Mulkens et al., 1996).

### Setup

The tasks were presented on a 144-Hz LCD screen (Eizo) with a distance of 68 cm from screen to eye and a 1,920 ×1,080 pixel resolution. Manual responses to the picture direction (left or right) were recorded with the Black Box ToolKit USB response pad (The Black Box ToolKit Ltd), where participants had to press the associated left or right button with the left or right index finger. To measure saccadic latencies in ms and decision times in ms, we used an EyeLink 1000 Plus with 1.000 Hz (SR Research, Inc).

### Task

**Fig. 4:**
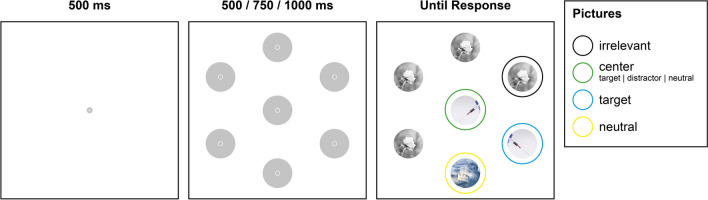
Example of the experimental setup. After a waiting period, the gray circles were replaced by pictures depending on the specific phobia. Participants were instructed to fixate on the center picture in the middle of the screen (green circle) as long as the circle was gray and to search for the target picture in the peripheral circle (blue circle) after the pictures appeared. The center could be a target, distractor, or neutral picture. When the target picture has been found, they should decide whether the picture orientation is left or right. The neutral picture (yellow circle) and the center picture should always be ignored. During one block, all pictures remained the same. (Color figure online)

In the visual search display, six gray circles (RGB: 196, 196, 196, 1.4° radius) were positioned around a center circle (RGB: 196, 196, 196, 1.4° radius) at a visual angle of 7.8° on a black background. Each circle contained a smaller gray circle with a 0.3° radius. Each trial started with a fixation point presented for 500 ms. The search display was presented after a waiting period of 500, 1,000, or 1,500 ms, randomly distributed and counterbalanced across all trials. In the search display, distinct pictures simultaneously replaced all circles (also 1.4° radius; see Fig. 4). The locations of distractors, targets, and neutrals pictures and the directions of the pictures were counterbalanced and presented in random order.

Participants were instructed to fixate on the center circle in the middle of the search display and only start searching when the distinct pictures were revealed according to the phobia subgroup (spider, snake, pointed objects). The center circle could change to a target (same picture condition; see Fig. 3A), distractor (similar picture condition; see Fig. 3 B), or a neutral (neutral picture condition; Fig. 3C) picture. Four peripherical circles were replaced with an irrelevant picture (a grayscale image of a flower; see Fig. 4, outlined in black), one peripherical circle was replaced with the target picture (see Fig. 4, outlined in blue), and one peripherical circle was replaced with the neutral picture (see Fig. 4, outlined in yellow). Target and distractor pictures were always either left- or right-oriented (picture direction).

After locating the target picture in the peripheral circle, participants were instructed to respond as quickly as possible to indicate whether it was oriented left or right, forcing them to process and not suppress it. All other features of the other peripherical circles and the center circle were to be ignored. The experiment consisted of five blocks, each with a different target, distractor, and neutral pictures, repeated once, resulting in ten blocks. Each block consisted of 42 trials, with the first ten trials in the first block considered practice and excluded from the data.

### Pictures

In general, pictures were selected based on four primary criteria: (1) clear object recognizability for threat-related pictures, (2) recognizable object orientation (left/right), (3) distinct object-background separation, and (4) consistent color spectrum (hue), luminance, and contrast within picture sets (one set for each of the five different blocks). Criterion 4, in particular, can influence the shared-feature effect since, depending on the target-distractor relationship, attention can be tied more or less, but it is much more important than deviations in luminance and hue still lead to shared feature effects (Wright, Boot, & Jones, 2015b). These criteria were applied to ensure consistency and clarity across all stimuli. One picture set (or search display) consisted of one target and one distractor, which were always threat related, one neutral-threat-unrelated picture, and four irrelevant pictures, which stayed the same across all conditions. There were five picture sets for each specific phobia.

### Statistical analysis

Saccadic latencies were calculated as the time between the presentation of the search display and the start of the first saccade, during which the saccade must leave the area of interest (AOI), which was 2.5° for each circle. Decision times were defined as the time between the first fixation of the target (fixation within AOI) and the button response. Thus, trials with no fixation of the target were removed from analyses. An eye movement was classified as a saccade if its distance exceeded 0.2° and velocity reached 30°/s. The start of the first saccade had to be at the center circle (94% of all trials started at the center circle). Trials with a latency of the first saccade faster than 90 ms^1^ (7.3% of all trials) and trials that included a blink before the first saccade (2.0% of all trials) were deleted. Further, trials with an incorrect manual response were excluded from the analysis (2.4% of all trials).

No participants were excluded. If not otherwise reported, median response times for each participant as a function of condition were calculated and used in all ANOVAs. For a manipulation check of whether the phobic group showed higher levels of fear towards the target and distractor pictures, we conducted two Welch’s tests (the assumption of equal variances was violated). For overall reaction times, saccadic latencies (H1 and H2a, H2b, and H2c), and decision times (H3), we used a 2 × 3 repeated-measures ANOVA, with frequential post hoc tests, each with the between-subject factor group (non-phobic vs. phobic) and within-subject factor picture condition (same, similar, and neutral). All tests were two-tailed, and a standard alpha value of .05 was used to determine whether the ANOVA and the post hoc tests suggested the null hypothesis could be rejected. We accounted for multiple testing by applying the Holm–Bonferroni method to calculate *p* values. Due to the law of large numbers, the normal distribution was assumed. We further tested for sphericity (*N* > 50). Because the sphericity assumption for saccadic latencies, χ^2^(2) = 50.83, *p* < .01, and decision times, χ^2^(2) = 11.50, *p* = .003, was violated, a Greenhouse–Geisser correction was applied. In the second step, disgust level should have been included as a control variable for H2a, H2b, and H3. However, because disgust ratings of the pictures and group were highly correlated (*r* = .85, *p* < .001), we did not include disgust as a control variable (problem of multicollinearity). All calculations were conducted using RStudio (Version 2023.06.1), R (Version 4.3.1; R Core Team, 2023) with the tidyverse package (Wickham et al., 2019), and JASP (Version 0.16.3; JASP Team, 2023).

## Results

**Fig. 5:**
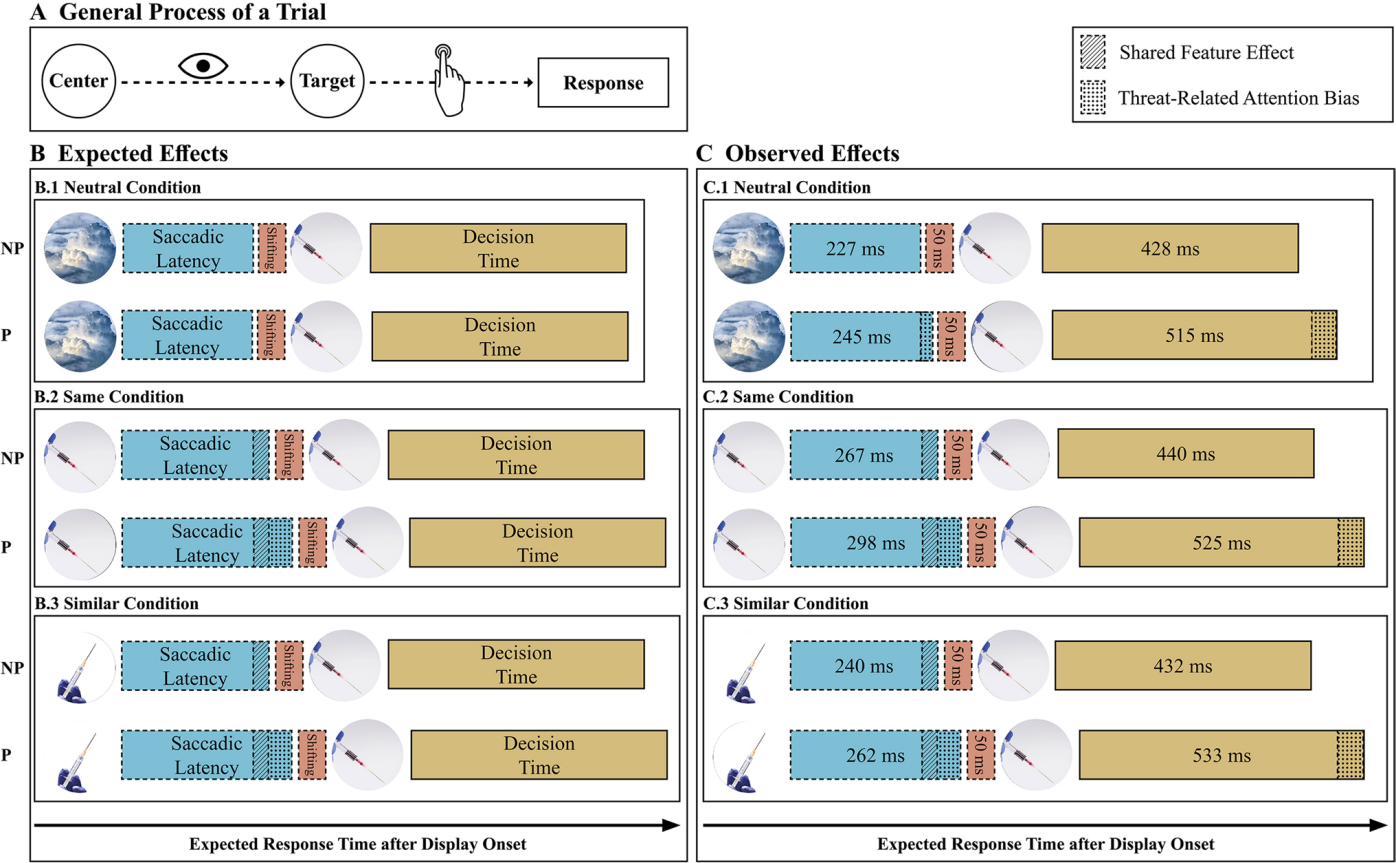
Expected (**B**) and observed (**C**) effects for the neutral (.1), same (.2), and similar (.3) picture conditions for the phobic (P) and non-phobic (NP) group depicted as a general process of a trial (see **A**). Overall reaction time is divided into saccadic latency (blue), shifting (red), and decision time (brown). The delay in disengagement due to the shared feature effect is indicated by the striped shading. Threat-related attention biases on the saccadic latency and decision-making are indicated by the dotted shading. (Color figure online)

The results are presented in five sections. In the first section, we conduct a manipulation check to ensure whether reported fear levels towards the experimental stimuli, as expected, differ in the phobic and non-phobic groups. In the second section, we test whether the type of specific phobia affected overall reaction times (time from display onset until button press). In the third section, we describe the descriptive statistics. In the fourth section, we examine whether there is a shared feature effect and whether the saccadic latencies (time from display onset until the start of the first saccade towards the target) in the three picture conditions differ by group. Last, we test whether decision times (time from landing on target until button press) in the phobic group are slower than the non-phobic group, independent of the picture condition. All hypotheses and the corresponding results are depicted in Fig. 5. The complete data, analysis, and experiment can be accessed online as additional material (https://osf.io/972f5/).

### Manipulation check

We conducted a repeated-measures ANOVA, with the between-subject factor group (phobic vs. non-phobic) and the within-subject factor fear rating of the pictures (target, distractor, neutral pictures) to test whether the phobic group indeed experienced stronger fear towards the presented pictures than the non-phobic group. The ANOVA revealed a significant interaction effect between the group and the fear rating, *F*(2,128) = 54.03, *p* < .001, η^2^ = .09. Post hoc tests showed that the two groups significantly differed in their fear ratings of the target (*M*_Diff_ = 2.99, 95% CI [2.18, 3.89], *p* < .001) and the distractor pictures (*M*_Diff_ = 2.91, 95% CI [2.01, 3.72], *p* < .001), with higher scores for the phobic group. There was no group difference for the neutral pictures (*M*_Diff_ = 0.73, 95% CI [−0.08, 1.55], *p* = .056).

### Type of phobia

The overall reaction times, *F*(2,63) = 1.40, *p* = .25, η^2^ = .04, as well as the saccadic latencies, *F*(2,63) = 3.042, *p* = .06, η^2^ = .09, and the decision times, *F*(2,63) = 0.974, *p* = .38, η^2^ = .01, did not differ between the three types of specific phobia (snakes, spiders, pointed objects). We therefore did not differentiate between types of specific phobia in the following analyses.

### Descriptive statistics

The descriptive statistics for the overall reaction time, saccadic latency, and decision time for all participants divided by group can be found in Table 1. The overall reaction time comprises the saccadic latency and the decision time. Note that adding the two values does not necessarily add up to the overall reaction time since other processes (i.e., shifting) were not considered. Additionally, we calculated the duration for shifting, which was about 50 ms across all conditions. Shifting time did not differ between groups. The descriptive statistics of all the pictures of the experiment can be found in the Appendix (Tables 2 and 3).

**Table 1:** Means of and standard deviation (in brackets) of overall reaction times, saccadic latencies, and decision times in ms aggregated across participants and separated by group and picture condition

	All			Phobic group			Non-phobic group		
	Picture condition			Picture condition			Picture condition		
	Same	Similar	Neutral	Same	Similar	Neutral	Same	Similar	Neutral
RT	852 (225)	839 (284)	793 (184)	922 (280)	921 (369)	855 (217)	782 (120)	756 (115)	731 (116)
SL	282 (56)	251 (36)	236 (34)	297 (68)	262 (42)	245 (41)	267 (35)	240 (23)	227 (22)
DT	483 (155)	483 (175)	472 (144)	525 (188)	533 (221)	516 (169)	440 (98)	432 (90)	428 (99)

*Note. N* = 66, RT = reaction time, SL = saccadic latency, DT = decision time

### Saccadic latency (h1, h2, and h3)

We conducted a repeated-measures ANOVA with the between-subject factor group (phobic vs. non-phobic) and the within-subject factor picture condition (same, similar, and neutral) to test H1, H2, and H3.

### Shared feature effect (h1)

The repeated measures ANOVA showed a significant main effect for picture condition, *F*(1.29,82.381) = 97.57, *p* < .001, η^2^ = .60. The effect size indicates a large effect. The Holm-adjusted post hoc analysis revealed significantly slower saccadic latencies for the same picture condition compared with the similar (*M*_Diff_ = 32 ms, 95% CI [23 ms, 39 ms], *p* < .001) and neutral picture condition (*M*_Diff_ = 47 ms, 95% CI [38 ms, 54 ms], *p* < .001). The similar picture condition further was significantly slower than the neutral picture condition (*M*_Diff_ = 15 ms, 95% CI [7 ms, 23 ms], *p* < .001). These results align with H1 and show a shared feature effect.

### Threat-related delayed disengagement (h2 and h3)

Testing H2, we found a significant main effect for the group, *F*(1,64) = 6.36, *p* = .014, η^2^ = .09, with a medium effect size. The phobic group was 24 ms (95% CI [5 ms, 43 ms], *p* < .014) slower than the non-phobic group, independent of the picture condition, confirming our assumptions.

**Fig. 6:**
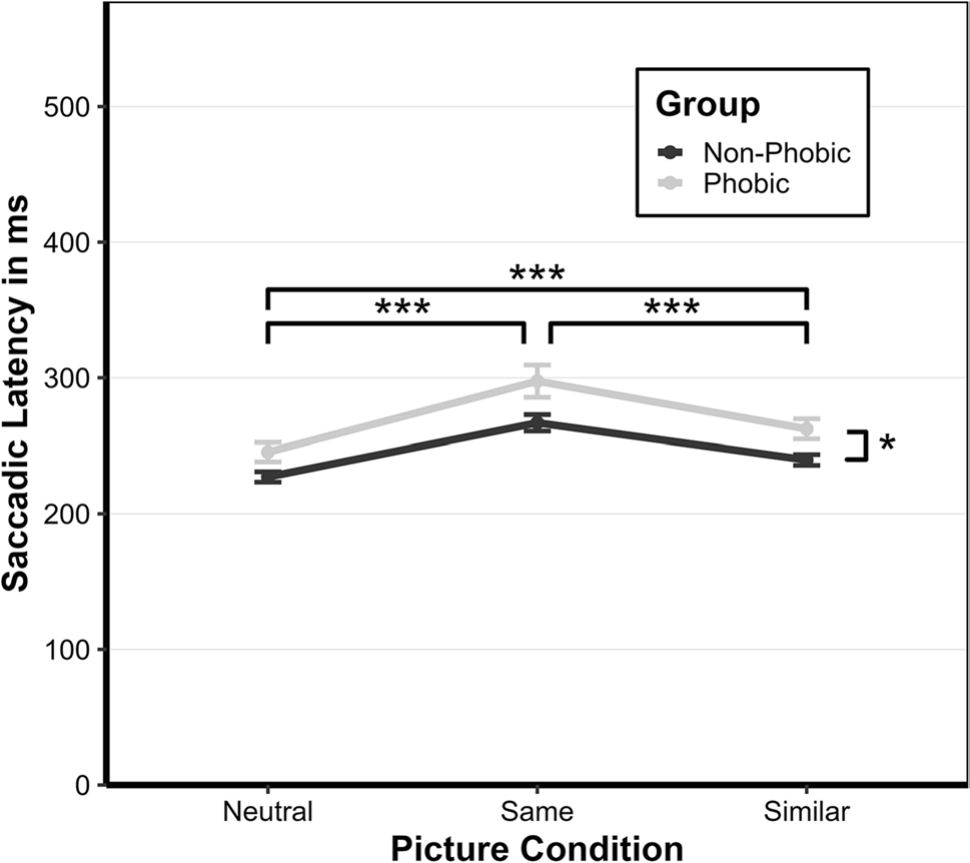
Saccadic latencies for picture condition and group. *Note*. Median response times for the saccadic latency in ms (calculated from individual participants’ medians) as a function of picture condition (neutral, same, or similar) and group (non-phobic or phobic). Error bars represent ±1 standard error of the median

The interaction effect between the picture condition and group (H3) was not significant and showed only a small effect size, *F*(1.29,82.38) = 1.76, *p* = .19, η^2^ = .03. Therefore, H3, that the delayed disengagement in the phobic group only occurs for threat-related stimuli, had to be rejected. Consequently, H3a, H3b, and H3c could not be tested because these require a significant interaction effect between the group and picture condition, and post hoc tests were not interpreted. Saccadic latencies are depicted in Fig. 6.

### Decision time (h4)

**Fig. 7:**
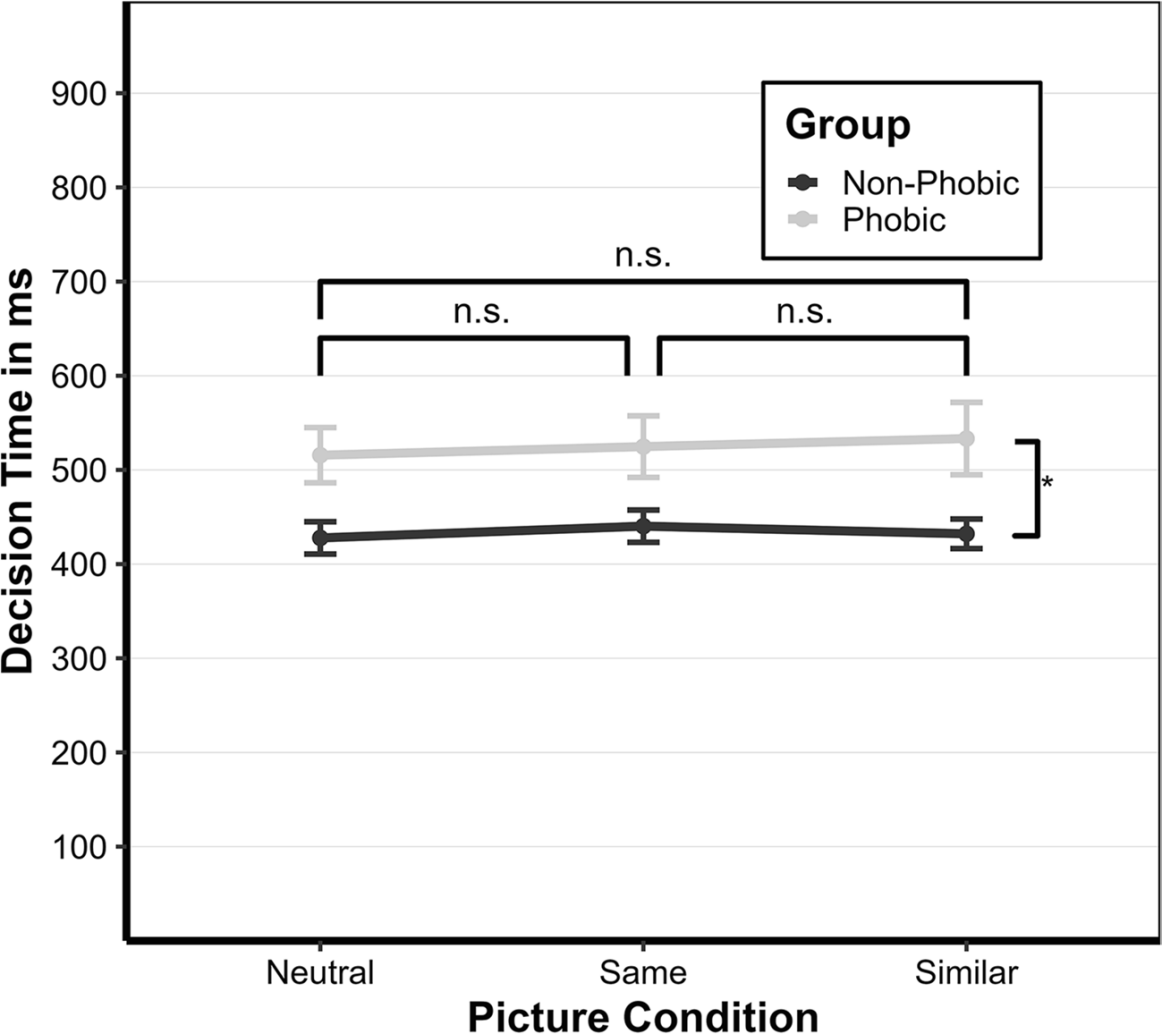
Decision times for picture condition and group. *Note*. Median response times for the decision time in ms (calculated from individual participants’ medians) as a function of picture condition (neutral, same, or similar) and group (non-phobic or phobic). Error bars represent ±1 standard error of the median

The repeated-measures ANOVA with the between-subject factor group (phobic vs. non-phobic) and picture condition as the within-subject factor (same, similar, and neutral) showed no significant main effect for the picture condition but a significant main effect with a medium effect size for the group, *F*(1,64) = 6.17, *p* = .016, η^2^ = .09, resulting in the rejection of H4. The phobic group was, on average, not faster, but 91 ms (95% CI [18 ms, 164 ms], *p* < .016, slower than the non-phobic group. No interaction effect was found. Decision times are depicted in Fig. 7.

## Discussion

The current study aimed to address the limitations of previous research in investigating threat-related attention bias, particularly the challenge of isolating specific attentional processes, as identified by Clarke et al. (2013). Therefore, we adopted a visual search paradigm using eye tracking previously described by Stefani et al. (2020). This paradigm integrates *overt* and *covert measures* of attention and, as we believe, can overcome some of the challenges of measuring threat-related attention bias. This experimental setup allows us to observe attentional processes separately. Specifically, engagement and shifting of attention were held constant across trials, and behavioral freezing was prevented for measuring delayed disengagement by avoiding uncertainty about possible behavioral alternatives by giving precise instructions. Therefore, disengagement of attention (saccadic latency) could be considered in isolation.

Our findings confirmed the shared-feature effect (H1), a phenomenon independent of any threat-related attention bias, which is characterized by delayed disengagement due to common features of an initially attended stimulus and a target stimulus. This effect was observed with complex stimuli (pictures), extending the findings of Stefani et al. (2020). Saccadic latencies averaged across all groups were significantly slower in the same (center and target picture topic are the same, e.g., the same picture of a spider) and similar (center and target pictures show similar topic, e.g., two pictures of different spiders) picture condition compared with the neutral (center and target picture topic is unrelated, e.g., a picture of a spider and a plant) picture condition. The same picture condition further resulted in a slower saccadic latency than the similar picture condition, supporting the assumption that the more features of the stimuli are shared, the stronger the delayed disengagement effect will be. This result is essential for future research on threat-related attention biases. Delayed disengagement effects might even occur when presenting stimuli that share the same topic (in our experiment, spider, snake, or pointed object). However, this slowing of saccadic latencies has nothing to do with anxiety or phobia. Future studies, therefore, should be careful in choosing their threat-related stimuli and their presentation.

In line with previous studies (see meta-analysis by Bar-Haim et al., 2007), our results indicated that phobic individuals exhibited 24 ms slower disengagement when viewing threat-related stimuli (H2). However, contrary to our expectations (H3a, H3b, H3c), we did not find an interaction effect between the group (phobic vs. non-phobic) and picture condition (same, similar, neutral), suggesting that saccadic latencies did not differ between the two groups in any of the three picture conditions. This finding raises questions about the mechanisms underlying these differences. Previous research has focused on two stages of the attentional process to explain the effects of threat-related stimuli (see also Armstrong & Olatunji, 2012): stimulus driven and goal driven. The stimulus-driven stage suggests that threat-related stimuli disrupt participants’ goals due to bottom-up processes, preventing rapid disengagement from a threat-related stimulus (Weierich et al., 2008). It is assumed that these individuals detect threat-related stimuli faster or that their detection thresholds for threatening stimuli are slower (Wiens et al., 2008). If our effects were raised from bottom-up processes, only saccadic latency in the same and similar conditions would have been affected. The goal-driven stage posits that attention biases emerge after threat detection, as threat-related stimuli hold the attention of high-anxious individuals for extended periods (Fox et al., 2001).

The ACT, proposed by Eysenck et al. in 2007, implies that the difficulty in disengaging attention from threat is related to the inhibition function of the central executive. This bias involves difficulty in inhibiting the initial processing of threats. While this imbalance in the attentional control systems of anxious individuals could primarily reflect stimulus-driven processes, ACT also suggests that general deficits in deactivating irrelevant anxiety-based goals in top-down processes play a role. Several studies further demonstrated deficits in attentional control related to non-threatening stimuli in anxiety (Ansari & Derakshan, 2011; Bishop, 2009). In fact, Derryberry and Reed (2002) found that difficulty disengaging attention from threats in anxiety was contingent on a more generalized deficit in attentional control. Shi et al. (2019) concretized and discussed that the efficiency, not the effectiveness (error rate), seems impaired.^2^ Our results support this view, as delayed disengagement was observed for both threat-related and neutral pictures, and the error rate did not differ between the groups.

Interestingly, we also observed slower decision times in the phobic group across all conditions. This was unexpected as neither engagement nor disengagement processes were involved once participants reached the target. However, Mogg et al. (2008) found similar results, which could be explained by behavioral freezing, as the participants had to remain on the target, which might have led to an orientation reaction or an overall impaired attentional control and inhibition of irrelevant information. In either case, the topic of the target (object of phobia) could not be efficiently ignored, resulting in slower decision times.

The generalizability of our findings to clinically relevant specific phobias remains an open question. Some studies have suggested that results from subclinical samples may not directly apply to clinical samples (Blicher & Reinholdt-Dunne, 2019; Blicher et al., 2020; Yiend et al., 2015). However, the implications of attentional control for clinical anxiety are intriguing and warrant further exploration. Derryberry and Reed (2002) claim that if good attentional control serves as a protective function, anxious individuals with poor control may be more vulnerable to clinical disorders, which is also supported by a study investigating attentional control as a moderator between attentional bias and PTSD (Clauss et al., 2022). Thus, delayed disengagement promotes prolonged attention to threats, amplifying the threat and increasing the likelihood of self-focused, ruminative, or catastrophic thinking (Derryberry & Reed, 1997). Thus, task-irrelevant threat-related stimuli influence attention control by reducing the goal-directed system.

## Limitations

Although our study shows that phobic individuals generally exhibit a delayed disengagement compared with non-phobic individuals independent of the presence of a threat-related stimulus, our study is also limited by certain aspects. One methodological constraint is the consistent use of threat-related target pictures. This was done to isolate engagement processes and prevent faster recognition of threat-related compared with neutral stimuli (Derryberry & Reed, 2002; Grafton et al., 2012). However, this approach implies that participants were always aware they would encounter a threat-related target, potentially influencing their responses (e.g., Ellenbogen & Schwartzman, 2009). It is also noteworthy that the comparison of the fear ratings for the neutral picture in the manipulation check (see section titled “Manipulation Check”) nearly reached significance with a *p* value of *p* = .056. Even though the groups did not differ significantly, descriptively, the phobic group rated the neutral pictures as more fear-inducing than the non-phobic group. This could indicate a spillover effect of a general fear network activation, which could be addressed in future research. In addition, all pictures were displayed before the commencement of the actual experiment as a part of the assessment of the fear and disgust ratings. This could have led to potential long-term priming effects in the phobic group (Rothermund & Wentura, 2014) that may have induced a feeling of fear or anxiety and activated the fear memory network prior to the experiment. Priming effects could also explain why, descriptively, the phobic group rated the neutral pictures as more fear-inducing than the non-phobic group. This could indicate a spillover effect of a general fear network activation, which could be addressed in future research. Further, clinical studies on exposure therapy show that habituation effects can occur after stimulus presentations, even during a single treatment session (Benito & Walther, 2015), which leads to a decrease in the response (Rankin et al., 2009). Thus, it is possible that all times were generally influenced and may have been slowed down by habituation processes prior to the experiment. We did not find a significant change in the responses over the 12 blocks during the experiment.

Furthermore, the shared-feature effect, which arises when two stimuli share common features, may have masked a potential threat-related delayed disengagement if these processes interact non-additively, especially since we believe that the shared-feature effect might be even pronounced in anxious individuals due to their impaired attentional control. Future research in threat-related attention bias should consider changing the picture condition blockwise to prevent influences of the threat on the neural stimuli. Another limitation is the reliance on self-reported phobia towards spiders, snakes, and pointed objects for group assignments. This subjective measure may be influenced by factors such as social desirability bias and current emotional states. In addition, we could not control for possible effects of disgust because the anxiety and disgust ratings of the pictures were highly correlated, resulting in the problem of multicollinearity. The fact that anxiety and disgust are inseparably involved in at least some specific phobia raises the question of what role disgust plays in anxiety-related attention bias (see also disease avoidance model; Davey, 1991). It is possible that threat-related attentional biases are not at all or not entirely caused by anxiety but by disgust (Knowles et al., 2019; Olatunji et al., 2017). Future research should address this issue in more detail. Furthermore, previous research has demonstrated that different species of animals, specifically snakes, can elicit varying levels of disgust and fear (Rádlová et al., 2019), which might have influenced our results. However, subsequent research has also shown that for people experiencing high levels of fear, the clear distinction dividing snakes into fearful and disgusting categories dissolves (Rádlová et al., 2020).

Finally, the generalizability of our findings to clinical populations with specific phobias or other anxiety disorders remains uncertain. Even though previous research has shown that anxiety-related attentional bias is observant both in subclinical and clinical samples, the magnitude of this bias differs (Bar-Haim et al., 2007). However, attentional biases may predict treatment response, suggesting that individual differences in these biases should be taken into account when tailoring interventions for anxiety disorders. By identifying and addressing specific attentional biases, clinicians can optimize their therapeutic strategies, potentially improving the efficacy of exposure therapies and other anxiety treatments (Barry et al., 2015). For example, cognitive bias modification (CBM) has proven to be a promising approach for directly changing these biases and reducing anxiety symptoms. By systematically altering attentional and interpretive biases, CBM can potentially alleviate anxiety by changing the way people process threatening information (MacLeod & Mathews, 2012). Future research, therefore, should aim to replicate these findings in clinical samples to enhance our understanding of attentional biases and how these contribute to anxiety disorders.

## Conclusion

Our findings not only replicated the shared-feature effect, a phenomenon seemingly independent of anxiety (see Stefani et al., 2020), but also demonstrated that individuals with specific phobia exhibit a pronounced delay in disengagement, reflected in slower saccadic latencies, regardless of whether the stimulus is threat-related or neutral. Furthermore, when viewing a threat-related picture, these phobic individuals took longer to respond to a simple task, such as determining a target’s left or right orientation. These results support the ACT (Eysenck et al., 2007), reinforcing the notion that individuals with phobic tendencies generally exhibit a deficiency in attentional control. They struggle to inhibit irrelevant task information, irrespective of whether the stimuli are threat-related (Bishop, 2009). This study, therefore, provides a nuanced understanding of attentional biases in individuals with self-reported specific phobia, marking an advancement in our comprehension of these complex processes.

## Data Availability

The datasets and materials as part of our study and/or analyzed during the current study are available at osf.io: https://osf.io/972f5/

## Footnotes

1 The instruction stated that the target was only allowed to be searched for (i.e., the fixation had to be released from the center) when the pictures were uncovered. With a latency of less than 90 ms, it had to be assumed that this instruction was not followed (see also Boot & Brockmole, 2010; Stefani et al., 2020).

2 Additional analysis using a repeated-measures ANOVA examined the error rate with the between-subject factor group (non-phobic vs. phobic) and the within-subject factor picture condition (same, similar, and neutral). No main effect for group, *F*(1.97,125.87) = 2.89, *p* = .060, η^2^ = .01, no main effect for picture condition, *F*(1,64) = 6.17, *p* = .016, η^2^ = .09, and no interaction effect, *F*(1.97,125.87) = 1.84, *p* = .163, η^2^ = .01, was found.

## Code availability

The generated code is available at osf.io: https://osf.io/972f5/
